# A Review of Non-Invasive Drug Delivery through Respiratory Routes

**DOI:** 10.3390/pharmaceutics14091974

**Published:** 2022-09-19

**Authors:** Yong-Bo Zhang, Dong Xu, Lu Bai, Yan-Ming Zhou, Han Zhang, Yuan-Lu Cui

**Affiliations:** 1 State Key Laboratory of Component-Based Chinese Medicine, Research Center of Traditional Chinese Medicine, Tianjin University of Traditional Chinese Medicine, Tianjin 301617, China; 2 Haihe Laboratory of Modern Chinese Medicine, Tianjin 301617, China

**Keywords:** scientometric analysis, nasal drug delivery, pulmonary drug delivery, nanoparticles, COVID-19

## Abstract

With rapid and non-invasive characteristics, the respiratory route of administration has drawn significant attention compared with the limitations of conventional routes. Respiratory delivery can bypass the physiological barrier to achieve local and systemic disease treatment. A scientometric analysis and review were used to analyze how respiratory delivery can contribute to local and systemic therapy. The literature data obtained from the Web of Science Core Collection database showed an increasing worldwide tendency toward respiratory delivery from 1998 to 2020. Keywords analysis suggested that nasal and pulmonary drug delivery are the leading research topics in respiratory delivery. Based on the results of scientometric analysis, the research hotspots mainly included therapy for central nervous systems (CNS) disorders (Parkinson’s disease, Alzheimer’s disease, depression, glioblastoma, and epilepsy), tracheal and bronchial or lung diseases (chronic obstructive pulmonary disease, asthma, acute lung injury or respiratory distress syndrome, lung cancer, and idiopathic pulmonary fibrosis), and systemic diseases (diabetes and COVID-19). The study of advanced preparations contained nano drug delivery systems of the respiratory route, drug delivery barriers investigation (blood-brain barrier, BBB), and chitosan-based biomaterials for respiratory delivery. These results provided researchers with future research directions related to respiratory delivery.

## 1. Introduction

Non-invasive drug delivery generally refers to painless drug delivery methods that provide alternative routes for the delivery of therapeutics via oral, nasal, pulmonary, ocular, or rectal [1]. Oral administration has been the most widely used way to treat diseases. However, some barriers affect the delivery of drugs to target sites, such as BBB and pulmonary barriers [2]. As a complex anatomical and physiological barrier, BBB selectively restricts the entry of substances into the brain; thus, effectively transporting drugs to the brain is a great challenge. Similarly, there are three significant barriers during pulmonary drug delivery. Mucociliary clearance is a mechanical barrier, mainly in the upper respiratory tract. Enzyme chemical barriers, such as peptidases and proteases, are responsible for protein and peptide degradation, and alveolar macrophage immunological barriers restrict the penetration of substances into the alveoli or further absorption into the systemic blood circulation [3].

Advances in anatomy and physiology provide advantageous features that make respiratory delivery an excellent route in drug delivery therapies. Novel drug formulations and devices are simultaneously developed to achieve more efficient drug delivery through the respiratory route. Nanoformulation is a particularly promising formulation for overcoming drug delivery barriers. Nanocarriers improve the efficiency of the drug and are promising viable formulations, making them important targets for research in preclinical and clinical practice [4]. In recent years, the study of respiratory delivery has increased markedly; however, theoretical reviews in respiratory delivery have rarely been summarized.

The scientometric analysis is an emerging method for literature or hotspot analysis. It has been widely used in scientific production and research trends in many disciplines and engineering fields [5]. Scholars use scientometric analysis for various reasons, such as revealing emerging trends in journal development or exploring the knowledge structure of specific fields in the existing literature [6]. Scientific analysis deciphers and maps mature fields’ accumulated scientific knowledge and evolutionary nuances by strictly making sense of large volumes of unstructured data. Therefore, scientometric research can promote the development of the field in novel and meaningful ways [7].

This study adopted scientometric methods to comprehensively analyze and process the research literature related to respiratory delivery. The research hotspots and general context in respiratory delivery will be concluded and discussed by analyzing the keywords of scientometric analysis. This review provides researchers with some meaningful insights and future directions related to the progress of respiratory delivery research.

## 2. Materials and Methods

### 2.1. Literature Search and Screening

**Figure 1:**
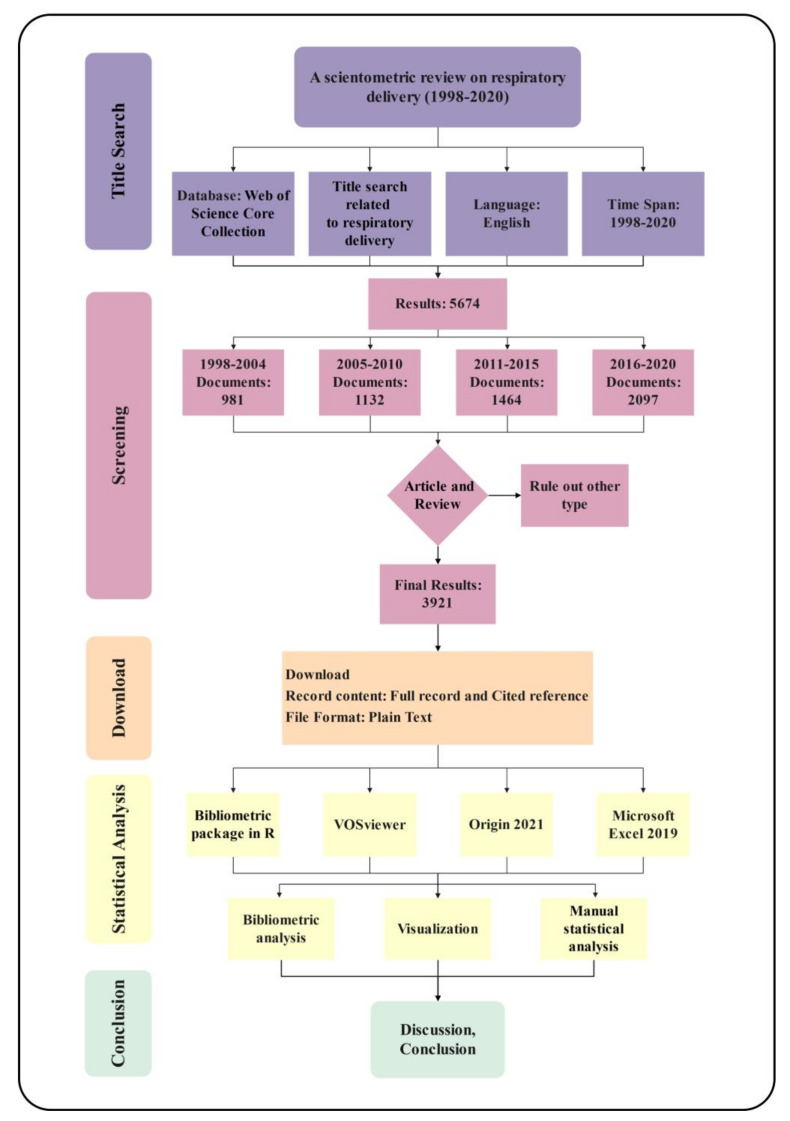
Flow chart of scientometric analysis of respiratory delivery.

All literature data were retrieved from the Web of Science Core Collection database on 11 October 2021. Respiratory delivery-related literature was collected using the search term “TI = (intranasal administration) OR TI = (intranasal delivery) OR TI = (nasal drug delivery) OR TI = (inhalation therapy) OR TI = (aerosol inhalation) OR TI = (nebulized inhalation) OR TI = (respiratory administration) OR TI = (respiratory drug delivery) OR TI = (inhaled drug) OR TI = (inhalation drug) OR TI = (atomization inhalation) OR TI = (nasal administration) OR TI = (inhaled medicines) OR TI = (inhaler therapy) OR TI = (pulmonary drug delivery) OR TI = (pulmonary administration) OR TI = (intratracheal administration) OR TI = (intrabronchial administration) OR TI = (dry powder inhalation)” in the advanced search. The flow chart of scientometric analysis of respiratory delivery is presented in Figure 1.

### 2.2. Data Analysis and Visualization

After filtering the literature, we downloaded the records, including full records and cited references from the Web of Science Core Collection database. The bibliometric package (Bibliometrix, Version 3.0.4) in R software (Version 4.0.5) was used to convert and analyze the bibliographic information of the publications. In addition, the map and cluster visualization of the keywords and institutions were visualized by VOSviewer software (version 1.6.16, Leiden University, Leiden, The Netherlands). Origin Pro 2021 (OriginLab, Northampton, MA, USA) was used to create graphics.

## 3. Results

### 3.1. General Information

Initially, 5674 articles published from 1998 to 2020 were identified. By screening the articles whose type was article or review, the final number of documents was 3921. From 1998 to 2020, 16,878 authors published 3921 articles on respiratory delivery in 1095 journals. The number of articles is essential to evaluate the field’s development trend and status quo. It was worth noting that the annual number of articles published on respiratory delivery increased from 103 in 1998 to 336 in 2020.

**Figure 2:**
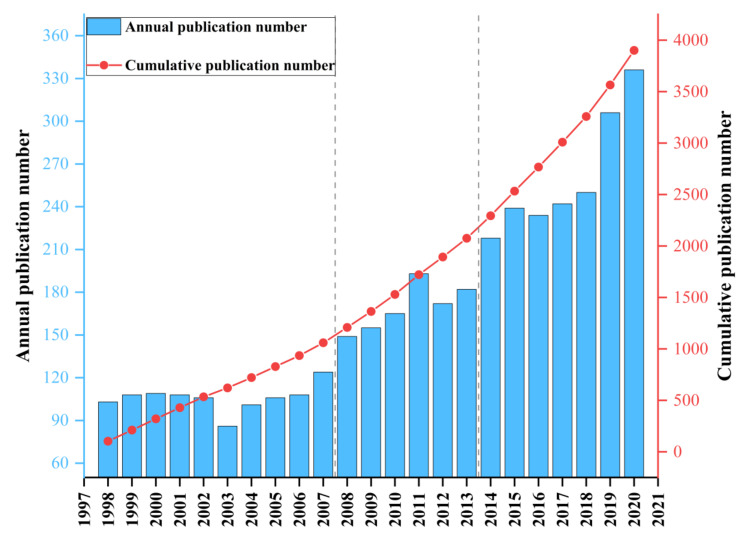
The number of papers published from 1998 to 2020 in respiratory delivery.

Based on the search results, the publication growth of respiratory delivery can be divided into three stages (Figure 2). In the first stage, the number of annual publications in this field was slow to increase. The outputs of annual publications on respiratory delivery research were less than 100 between 1998 and 2007. In the second stage, the number of annual publications rapidly grew. From 2007 to 2014, the output showed a steady rising growth from 124 to 218. In the third stage, the annual number of documents rapidly increased for the second time. Between 2015 and 2020, the production exceeded 230 and peaked at 336 in 2020. Overall, the cumulative number of published articles shows an increasing trend over the years, suggesting growing attention from researchers.

### 3.2. Keywords Analysis

**Figure 3:**
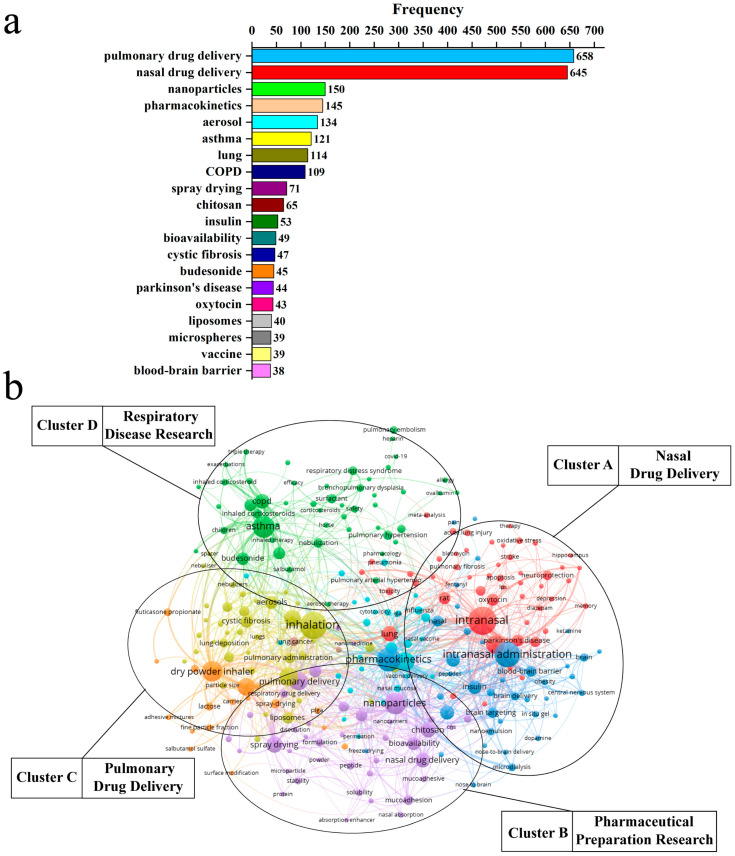
Keywords map. (**a**) Top 20 keywords with the highest frequency. (**b**) Keywords co-occurrence network. Each node’s size indicates the keyword’s frequency. Links connecting two nodes represent a co-occurrence relationship between two keywords.

According to scientometrics theory, keyword analysis can reveal research hotspots and trends [8]. Therefore, the hot topic and future development directions were analyzed using keywords frequency, which is helpful for researchers who wish to quickly grasp the development trends of scientific research. We analyzed and ranked author keywords from 3921 papers. The top 20 keywords with a high frequency are shown in Figure 3a. The research hotspots in respiratory delivery mainly included research on respiratory diseases such as chronic obstructive pulmonary disease (COPD), the development of advanced preparations such as nanoparticles, the research on drug delivery barriers such as the BBB, and chitosan-based biomaterials for respiratory delivery.

A co-occurrence analysis of keywords can help to classify the main knowledge structures and hotspots. We used the VOSviewer software to generate the high-frequency keyword co-occurrence network through literature data analysis (Figure 3b). It was found that keywords were divided into four clusters: (1) nasal drug delivery: BBB, brain targeting, nasal vaccine, brain delivery, nose to brain delivery, etc. (Cluster A); (2) pharmaceutical preparation research: nanoparticles, chitosan, nanocarriers, formulation, etc. (Cluster B); (3) pulmonary drug delivery: dry powder inhaler, inhalation, pulmonary delivery, lung deposition, aerosols, etc. (Cluster C); and (4) respiratory disease research: asthma, COPD, COVID-19, pulmonary arterial hypertension, etc. (Cluster D). Additionally, the analysis results of institutional contribution (Figure S1a), coun-try/region cooperation (Figure S1b,c), high-yield journals (Table S1), and high-ly-cited papers (Table S2) are presented in supplementary materials.

## 4. Discussion

### 4.1. Analysis of Scientometric Results

To gain insight into respiratory delivery, we analyzed 3921 articles from the last two decades. According to the increasing number of publications, the field of respiratory delivery is still in a continuous development trend. Although slightly fluctuating, the number of articles doubles almost every five years. After long-term accumulation of knowledge, respiratory delivery has entered a period of rapid development. Publications in the last five years accounted for nearly half of the total articles on respiratory delivery from the past 20 years.

According to the results of the keywords frequency analysis, several articles on pulmonary and nasal drug delivery in respiratory delivery have appeared in the past two decades. The results of keyword co-occurrence analysis indicated that respiratory delivery has been extensively used to treat various diseases. This trend may be due to the superiority of respiratory administration over traditional drug administration (i.e., oral or intravenous administration). For example, nasal drug delivery has the characteristics of non-invasiveness, rapid onset, and small dose, while pulmonary drug delivery has the advantages of rapid drug absorption, elimination of first-pass metabolism, and fewer adverse reactions. These two drug delivery routes can show sound curative effects regarding local and systemic administration [9].

In addition, Cluster 4 showed that multiple drug delivery systems were designed for administration through the respiratory route. The most studied nanoformulation is represented by nanoparticles, the largest node in Cluster 4. Nano-based drug delivery strategies offer various site-specific and targeted delivery advantages for disease treatment. Nano sized drugs avoid mucociliary clearance and are not phagocytosed by alveolar macrophages, increasing therapeutic agents’ uptake in the respiratory system [10]. Additionally, nano drug delivery systems can improve nose-to-brain delivery by protecting encapsulated drugs from biological and chemical degradation and by transporting P-gp efflux proteins across extracellular spaces [11]. Several nano drug delivery systems with specific biochemical properties have been designed to achieve personalized and targeted therapy [12].

**Figure 4:**
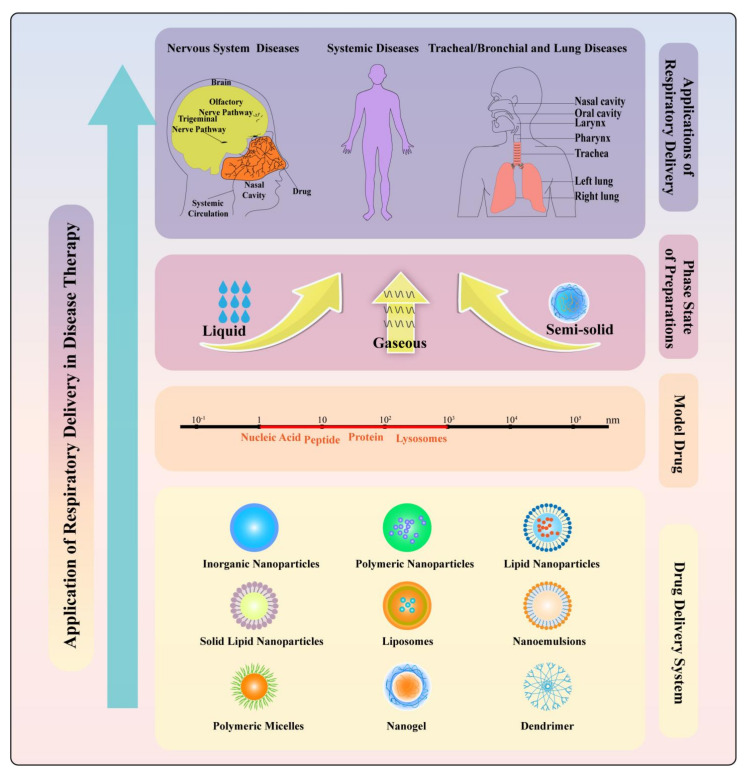
Application of respiratory delivery in disease therapy.

**Figure 5:**
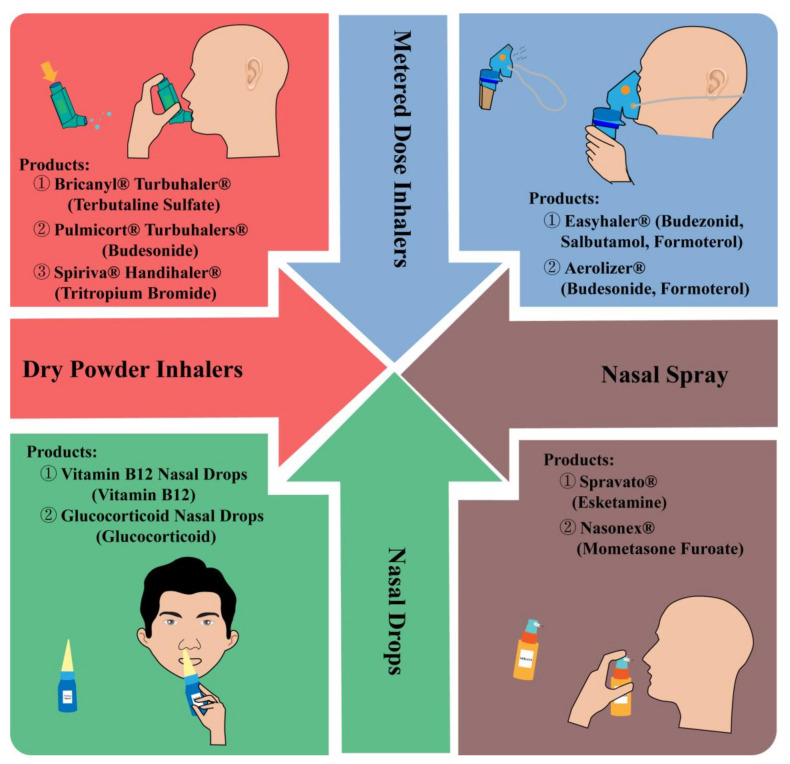
Different products for respiratory delivery [13,14,15,16,17,18].

**Figure 6:**
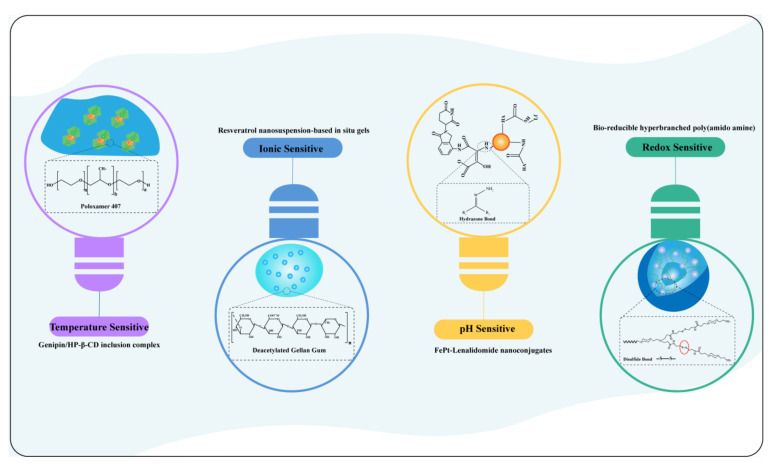
Stimuli-sensitive preparations for respiratory delivery. Purple cluster: temperature-sensitive formulation, genipin/HP-β-CD inclusion complex; blue cluster: ionic-sensitive formulation, resveratrol nanosuspension-based in situ gels; orange cluster: pH-sensitive formulation, FePt-Lenalidomide nanoconjugates; green cluster: redox-sensitive formulation, bio-reducible hyperbranched poly (amido amine) [19,20,21,22].

Therefore, based on scientometric analysis, the advantages and challenges of respiratory delivery, the application of respiratory delivery in disease therapy (Figure 4), different products of respiratory delivery (Figure 5), and stimuli-sensitive preparations for respiratory delivery (Figure 6) were discussed.

### 4.2. Non-Invasive Drug Delivery

A significant challenge in achieving therapeutic success is the efficient delivery of drugs. Due to pain, infection risk, high cost, and low patient compliance, parenteral injections are not the preferred method of administration. Furthermore, concerns about needle disposal hinder parenteral administration [23]. There are significant advantages to non-invasive drug delivery over injection, including a decreased risk of needle sticks, a low risk of infection, and an increased level of patient compliance with treatment [24,25].

However, molecular size, hydrophilicity, low permeability, chemical or enzymatic instability, and low permeability of therapeutics pose formidable obstacles to developing non-invasive drug delivery systems [26]. Many small molecules and almost all biologics do not possess the ideal physicochemical properties for good absorption from mucosal surfaces or the skin. Various formulation strategies have been proposed to overcome these problems. Innovative nanoformulations with controllable particle sizes and surface modifications have been developed to improve target selectivity, systems half-life, and bioavailability of drugs. Nanotechnology plays a vital role in developing non-invasive drug delivery systems to improve drug clinical outcomes [27].

Non-invasive drug delivery mainly includes oral, nasal, pulmonary, rectal, and transdermal routes. Oral administration is the most used drug delivery strategy due to its convenience. Still, it faces the absorption and degradation of drugs in the gastrointestinal tract due to the acidic environment and microorganisms [28]. Transdermal administration can eliminate first-pass metabolism and maintain sustained drug release, thereby reducing dosing frequency. The stratum corneum, the outermost part of the skin that contains dead keratinocytes and lipids [29], acts as a drug barrier because it limits the absorption of large molecular weight and hydrophilic molecules. Intestinal irritation and poor patient compliance are some of the problems associated with rectal administration.

Respiratory delivery (nasal and pulmonary drug delivery) has unique advantages compared to other non-invasive drug delivery routes. Nasal drug delivery can bypass hepatic first-pass metabolism, is effective, and patient-friendly regarding self-medication. Moreover, it allows drugs to bypass the BBB and be directly delivered to brain tissue or cerebrospinal fluid via olfactory neurons [30]. In the lungs, the size of the surface area (about 80–140 m^2^), thin alveolar epithelium (0.1–0.5 mm), and ample blood supply contribute to rapid and high drug absorption [31]. All these advantages make nasal and pulmonary routes suitable for local and systemic drug delivery.

### 4.3. Therapy for Central Nervous System Disorders

Despite an increase in the prevalence of neurological and psychiatric disorders such as Parkinson’s disease, Alzheimer’s disease, brain tumors, and depression at the global level, drug molecules are still not effectively delivered to the CNS [32]. It is possible to directly administer drugs into parenchyma or cerebrospinal fluid via intraparenchymal or intrathecal infusions, respectively, but these routes of administration are invasive and unsuitable for chronic treatment [2]. Furthermore, delivering diagnostic agents or therapeutic drugs with non-targeted may also severely damage neurons and glial cells. Therefore, there is an urgent need for new avenues to deliver therapeutic drugs for neurological diseases [33]. More and more researchers have paid attention to treating CNS diseases by nasal drug delivery. However, the precise drug delivery mechanism of the nasal cavity to the CNS is not fully understood. Several reports demonstrate that nose-to-brain drug delivery is mainly mediated by olfactory and trigeminal nerve pathways located at the roof of the nasal cavity [34]. The functional pathway through which drugs pass into the CNS from structures deep in the nose innervated by the cranial nerve is called “nose-to-brain” transport [35]. In addition, the treatments of Parkinson’s disease [36], anxiety [37], and migraines [38] have reported promising results for delivering CNS therapeutics through different inhaler techniques.

#### 4.3.1. Bypass the Blood-Brain Barrier

The CNS is protected by a highly regulated complex structure called the BBB [39]. The BBB is composed of the capillary endothelium, pericytes, and the astrocyte foot processes [40]. Specifically designed endothelial cells of the BBB do not have fenestrations, and they possess extensive tight junctions that prevent hydrophilic and polar compounds as well as molecules with high molecular weight from entering the CNS from the bloodstream [41]. Astrocytes, which surround cerebral capillaries, also prevent passive diffusion through the cell membrane to minimize the uptake of extracellular substances [42]. Although the structure of the BBB is vital to keep harmful substances out, it is also a significant obstacle to the pharmacological treatment of CNS and mental diseases [43].

The nose-to-brain route provides a practical, non-invasive way to bypass the BBB and delivers therapeutic agents to the brain. A variety of drug delivery systems, such as nanogels [44], nanoparticles [45], and solid lipid nanoparticles [46], have been studied to deliver drugs to the CNS through intravenous administration, which can cross BBB [47]. However, intravenous administration is not suitable for major depressive disorder, Parkinson’s disease, and other diseases that need daily administration. Nasal drug delivery has the following advantages: (1) about 98% of small molecules and almost all large proteins and genes cannot pass through the BBB [48], but intranasal administration can bypass the BBB and deliver drugs directly to the brain; (2) the nasal cavity has a lot of strengths such as high surface area, high permeability, high vascularization, and the nose-to-brain pathway; and (3) the nose-to-brain route avoids first-pass metabolism in the gastrointestinal tract and liver, thereby avoiding most drug inactivation.

Many pharmaceutical preparations have been used to improve penetration through the physiological barrier of the nasal cavity. As a drug preparation method, nanotechnology combined with the nasal drug delivery route is expected to provide an effective drug delivery system [21,49]. Currently, by loading a drug that is poorly distributed in the brain into the nanocarrier system, good interactions with the nasal mucosa can be achieved to prolong the residence time and ultimately produce higher drug concentration in the brain parenchyma. Such a nanocarrier can be modified with targeting ligands to preferentially bind to receptors or transporters expressed at the BBB to enhance brain selectivity and permeability [50]. In addition, by crossing the barrier through the process of cell transcytosis, this system can be further used for effective drug delivery [51]. Among them, nanoparticles are one of the most researched drug delivery systems, ranking as the third most frequent keyword. Since nanoparticles can protect encapsulated drugs from degradation, chemicals, and P-gp efflux, proteins are extracellularly delivered, thus improving the nose-to-brain drug delivery [25].

#### 4.3.2. Treatment of Parkinson’s Disease

Parkinson’s disease (PD), a neurodegenerative disorder, affects approximately 1–2% of the population over the age of 65 [52]. It is characterized by the loss of dopaminergic receptors in the nigrostriatal and mesolimbic systems in the brain [53]. As α-synuclein accumulates and dopamine levels are depleted, it develops into the dysfunction of daily tasks. Symptoms of the disease include movement disorders, tremors, and difficulty walking due to a loss of balance and coordination. Treatment for PD involves increasing dopamine levels in the brain or reducing the concentration of dopamine inhibitors. Drugs such as levodopa and carbidopa are converted into dopamine when delivered to the brain.

Combining nanotherapeutics with nasal drug delivery promises to bypass the BBB for targeted therapy to the brain. This approach has been effective in treating a variety of neurodegenerative diseases in previous studies. Monoamine oxidase B (MAO-B) inhibitors are an established therapy for PD and work in part by blocking the MAO-catalyzed metabolism of dopamine in the brain [54]. Selegiline and rasagiline are two MAO-B inhibitors commonly used in the clinic. Sridhar et al. synthesized selegiline-loaded chitosan nanoparticles by ionic gelation [55]. Compared to oral administration, selegiline concentrations in the brain and plasma were 20- and 12-fold higher, respectively, after intranasal administration, greatly reducing the dose and the side effects of selegiline. Furthermore, C_max_ in the brain after intranasal nanoparticle administration was significantly higher than that after intranasal thermosensitive gel and plain solution administration. This suggested that chitosan was a biodegradable permeation enhancer that increases the nasal mucosal penetration of selegiline nanoparticles. Rasagiline has advantages over selegiline because its metabolites do not include potentially toxic amphetamines [56]. Mittal et al. used the ionic gelation technique to prepare rasagiline-loaded chitosan glutamate nanoparticles (RAS-loaded CG-NPs) [57]. After intranasal and intravenous administration, the biodistribution of RAS formulations in mice brains and blood was performed using the HPLC. Intracerebral drug concentrations were significantly always elevated in nasally administered CG-NPs. The results showed that the brain bioavailability was significantly improved following intranasal administration of RAS-loaded CG-NPs, which may represent an achievement of direct nose-to-brain targeting in PD treatment.

#### 4.3.3. Treatment of Alzheimer’s Disease

Alzheimer’s disease (AD) is a degenerative brain disease characterized by memory loss and associated cognitive impairments, including poor judgment and decision-making, language impairment, loss of temper control, and emotional disturbances [58]. AD initially occurs with the loss or destruction of neurons involved in the brain’s cognitive function. Neurons in other brain parts are gradually destroyed, making it difficult to perform essential body functions, such as walking and swallowing. Eventually, the brain will lose its function completely. Although genetics, age, and environmental factors influence this disease, the etiology of AD is not fully understood. Drugs that FDA has approved for AD treatment act as cholinesterase inhibitors and include donepezil, rivastigmine, galantamine, and tacrine [59]. Currently, most approved drugs are orally administered in tablet form. Still, they suffer from poor absorption from the digestive tract, difficulty reaching the brain, and a lack of effectiveness at recommended doses. There are several ways to deliver specific drugs to the brain, one of which is the nasal route [60].

Tacrine hydrochloride (THA) is an FDA-approved drug for AD treatment with a short half-life. It is a reversible acetylcholinesterase inhibitor that enhances the deficiency of brain cholinergic neurotransmission and prevents the degradation of neurotransmitters by increasing the level of acetylcholine in the brain [61]. Jogani et al. investigated a microemulsion delivery system to enhance nasal-brain transport of THA. Additionally, a mucoadhesive agent (Carbopol 934P) was added to prolong the contact time of the formulation in the nasal cavity and enhance THA absorption in the brain, reducing systemic distribution and side effects. The prepared formulation exhibited the mean globule size < 27 nm and zeta potential < −20 mV, and the addition of a mucoadhesive agent further negatively contributed to the system. The biodistribution of the THA solution and its formulation after intravenous and intranasal administration was assessed using 99mTc as a marker. Results showed that nasal drug delivery enhanced brain selectivity and accumulation of THA over intravenous administration [62]. In another study, Qian et al. conducted a pharmacokinetic and pharmacodynamic study of HLS-3, a tacrine dimer with high anti-acetylcholinesterase activity, for the treatment of AD. The results showed that intranasal administration of HLS-3 had similar central effects and fewer peripheral adverse effects at a much lower dose than oral tacrine, suggesting that intranasal administration of HLS-3 might be a potential therapy for AD treatment [63].

#### 4.3.4. Treatment of Depression

Depression is a common mental illness that affects a person’s thoughts, behaviors, feelings, and circadian rhythms. Pathological causes of depression include chemical imbalances in the brain, decreased energy metabolism, and altered hormone levels [64]. According to the serotonin hypothesis, depression results from dysfunctional serotonergic activity leading to reducing serotonin levels in the brain. Selective serotonin reuptake inhibitors (SSRIs) such as paroxetine, vilazodone, and fluvoxamine are first-line medications for patients with depression [65]. However, the approved oral antidepressants have reduced bioavailability due to first-pass metabolism. The time it takes for a drug to reach its saturation point is often prolonged, resulting in delayed and reduced efficacy. In addition, due to low bioavailability, higher doses need to be ingested, leading to an increased incidence of side effects [66]. Furthermore, the therapeutic effect is also limited due to the presence of the BBB.

Nasal drug delivery is an attractive therapeutic strategy and has been used to treat depression. Ketamine, an intranasal antidepressant, has been studied in multiple clinical trials. As an antidepressant, there are several hypotheses for the mechanism of action of ketamine, including synaptic or GluN2B-selective extra-synaptic N-methyl-D-aspartate receptor (NMDAR) inhibition, inhibition of NMDARs localized on GABAergic interneurons, inhibition of NMDAR-dependent burst firing of lateral habenula neurons, and the role of α-amino-3-hydroxy-5-methyl-4-isoxazole-propionic acid receptor activation. These preclinically proven ketamine mechanisms of action are not mutually exclusive and may act synergistically to exert the drug’s antidepressant effects [67,68]. In March 2019, FDA approved intranasal esketamine (the S (+) enantiomer of ketamine) for the treatment of treatment-resistant depression [69]. It was the first antidepressant in the form of a nasal spray, and the therapeutic effects of intranasal esketamine were observed after 4 h from application [70]. The rapid onset of action is a considerable advantage of esketamine compared with conventional antidepressants.

Intranasal administration is one of the easier options for delivering molecules to the brain region. Antidepressants like venlafaxine increase synaptic neurotransmitter levels that are diminished due to depression [71]. Due to its short elimination half-life and inherent hydrophilicity, it requires frequent administration to maintain its therapeutic concentration and its penetration into the brain needs to be improved. Using venlafaxine-loaded alginate nanogels, Hague et al. showed increased drug permeation through the isolated porcine nasal mucosa. This was attributed to the positively charged amino groups on alginate and the negatively charged sialic acid on the cell membranes. In vivo studies of rats showed that the drug had a higher brain to blood ratio after intranasal administration, revealing that the drug bypassed the BBB and was transported directly to the brain via the nose. Intranasal administration produced a significantly higher brain concentration of the drug (743 ng/mL; t_max_ = 60 min) than intravenous administration (383 ng/mL; t_max_ = 30 min). In another study, Haque et al. designed antidepressant nanoparticles containing venlafaxine-loaded alginate chitosan (VLF AG-NPs) for the nose-to-brain treatment of depression. The antidepressant activity of VLF AG-NPs was evaluated by the forced swim test. It showed a significant improvement in behavioral analysis parameters, including swimming, climbing, and immobility, which was observed after intranasal administration of VLF AG-NPs [72].

#### 4.3.5. Treatment of Glioblastoma

In adults, malignant brain tumors are devastating diseases with high morbidity and mortality. Among children, they are the second leading cause of cancer-related death [73]. Currently, there are no effective therapies, mainly due to high tumor heterogeneity, chemoresistance, and difficulties imposed by BBB. It is urgently needed to develop new treatment options for glioblastoma, which currently undergoes surgery, radiation therapy, and chemotherapy concurrently [74].

Various types of biodegradable and biocompatible polymers have been widely studied, which can prevent toxic and side effects and simultaneously achieve the goal of sustained and controlled release. Chitosan, the 10th highest frequency keyword in the keyword frequency analysis, is the only polycationic polysaccharide extracted from biological sources and is widely used in nano drug delivery systems. Chitosan can increase the residence time of drugs in the olfactory area, reduce mucus clearance, and open the tight junctions between epithelial cells, thereby enhancing the penetration of drugs through mucosal membranes. Galectin-1 (Gal-1), a natural galactose-binding lectin, is overexpressed in glioblastoma multiforme (GBM). To inhibit Gal-1 in GBM, Woensel et al. prepared a highly concentrated suspension of small interfering RNA (siRNA)-loaded chitosan nanoparticles to deliver siRNA to the CNS via nasal drug delivery. The chitosan nanoparticles did not affect the effectiveness of siRNA molecules and rapidly delivered siRNA to murine and human GBM cells, thereby reducing tumor cell migration. Following intranasal administration in healthy mice, it was observed to rapidly spread into the nasal mucosa and further into the olfactory bulbus and the hindbrain [75]. Cetuximab (CET) is an anti-epidermal growth factor receptor (EGFR) monoclonal antibody developed for brain tumor targeting because CET specifically binds with high affinity to EGFR. EGFR is simultaneously overexpressed in most brain tumors but not expressed in normal tissues [76]. Ferreira et al. [77] prepared poly (lactic-co-glycolic acid) (PLGA) and oligomeric chitosan (OCS)-based mucoadhesive nanoparticles to co-deliver α-cyano-4-hydroxycinnamic acid (CHC) and CTX into the brain by nasal drug delivery. Stable nano sized particles (213–875 nm) with high positive surface charge (+33.2 to +58.9 mV) and entrapment efficiency (75.69 to 93.23%) were produced by emulsification/evaporation technique and further combined with the CTX. The CHC-loaded NPs exhibited high cytotoxicity against different glioma cell lines (U251 and SW1088). Analysis of chicken chorioallantoic membranes demonstrated the anti-angiogenic activity of CHC-loaded NPs. In conclusion, this nano drug delivery system can potentially be a new therapeutic alternative for glioblastoma therapy.

#### 4.3.6. Treatment of Epilepsy

Epilepsy is a neurological disorder characterized by recurrent seizures that affect people of all ages, genders, races, and geographic locations [78]. Nearly 50 million people are infected worldwide, with a prevalence rate of 5–10 per 1000 people and more than 0.5% of the global disease burden [79].

Antiepileptic drugs, including phenobarbital, phenytoin, felbamate, topiramate, vigabatrin, and gabapentin, are usually taken orally in the form of tablets, capsules, solutions, and suspensions [80]. Commercial antiepileptic doses are ineffective for about one-third of epileptic patients [81]. Drug resistance may result from a drug not reaching its target area or from a lower concentration of the drug in the brain. Researchers have used nanoformulations to deliver antiepileptic drugs to receptor sites via intravenous, oral, nasal, and transdermal routes [82]. Jain et al. formulated amiloride-loaded mucoadhesive nanoemulsions for nose-brain administration. The mucoadhesive nanoemulsions had no toxicity to the sheep nasal mucosa and were determined to be safe after intranasal administration of antiepileptic drugs. The developed formulation also needs to be pharmacokinetically and pharmacodynamically evaluated [83]. Gonçalves et al. studied a thermoreversible gel loaded with levetiracetam and administered it to mice in an aerosolized form. Pluronic F-127 and Carbopol^®^ in the formulation helped form a thermosensitive gel with adequate structural characteristics so that it can act as a liquid aerosol gel in the nasal mucosa. The absolute intranasal bioavailability was 107.44%, indicating a high proportion of the drug systemically absorbed. Histopathological examination of lung tissue after repeated administration by intranasal route showed that the preparation caused no toxicity or structural damage. Although further exploration is required, these nonclinical studies offer new hope for epilepsy treatment [84].

### 4.4. Therapy for Tracheal/Bronchial and Lung Diseases

Tracheal or bronchial and lung diseases are important causes of death globally, including cough, common cold, and more severe diseases like pulmonary hypertension, lung cancer, and COPD [85]. Due to the limitations of conventional administration routes and existing treatment methods, the pulmonary administration route has attracted widespread attention. It has become an important research area for effective therapeutic interventions for respiratory diseases [86]. Due to the large surface area of the respiratory endothelium, the elimination of the first-pass metabolism, and small dosages, pulmonary drug delivery has shown enormous potential and has attracted significant attention in pulmonary disease treatment [87]. Compared to oral or parenteral routes, even if the dose is reduced, it can take effect quickly and help to minimize adverse reactions. Pulmonary drug delivery can also deliver high concentration biologics to the lungs, and can be effectively used for respiratory infections, lung cancer, and asthma [88]. We have summarized some of the FDA-approved inhalation products in Table 1.

#### 4.4.1. Treatment of COPD

COPD is a progressive, debilitating respiratory disease that affects millions worldwide [90]. In COPD, the airway wall loses its elasticity, so it collapses and gets stuck, causing patients to encounter problems during expiration. COPD is a group of two diseases: (1) emphysema with parenchymal destruction and loss of alveolar septa and (2) chronic bronchitis. Emphysema typically refers to the loss of elastic properties in the lungs, while chronic bronchitis involves severe inflammation in the lungs, leading to excessive mucus production [91]. Several factors cause COPD, including smoking, environmental exposure, and genetic predisposition. Long-acting bronchodilators, beta-agonists, anticholinergics, and corticosteroids are the choices for treating COPD [92,93].

It is exciting to think about delivering nanoparticles as aerosols. Many living cell components are constructed at the nano level, such as ribosomes, membrane transporters, and receptors [94]. Nanoparticles fall within the same size range of biological entities, so they can easily interact with molecules on the cell surface and within the cell. In addition, drugs deposited within the lungs in the form of nanoparticles are more likely to escape from the clearance mechanisms by the lung defense system than in the form of microparticles [95]. Therefore, drug-loaded nanoparticles may effectively deliver drugs to the epithelium while avoiding unnecessary mucociliary clearance. Researchers have prepared and characterized various nanoparticles for treating diseases like COPD. Geiser et al. used the Scnn1b-transgenic mice model to recapitulate the key features of human COPD and used the manufactured gold nanoparticles (AuNPs) to study the uptake and localization of macrophages. Inhaled AuNPs rapidly bound to mice alveolar epitheliums; delayed macrophage uptake of nanoparticles was observed to promote the in-depth translocation of nanoparticles in Scnn1b-transgenic mice, indicating that such nanoparticles could be an effective method to treat COPD [96]. In another study, Mohamed et al. formulated nanocomposite microparticles (NCMPs) of microRNA (miR-146a) for dry powder inhalation using L-leucine and mannitol. The NPs size (409.7 ± 10.05 nm) was still suitable for targeting respiratory bronchioles after spray drying, and the miR-146a activity was still preserved. Spray drying of NCMPs with retained miR-146a activity demonstrated potential gene therapy for COPD via dry powder inhalation [97].

#### 4.4.2. Treatment of Asthma

Asthma is a prevalent global disease that affects more than 300 million people, and morbidity is increasing [98]. Generally, asthma is divided into five groups based on the primary irritants that cause acute episodes: allergic asthma, non-allergic asthma, occupational asthma, aspirin-induced asthma, and infant asthma [99]. Chest tightness, airway inflammation, shortness of breath, and cough are the main symptoms of asthma. The immuno-histopathological manifestations of asthma are infiltration of inflammatory cells such as lymphocytes, neutrophils, eosinophils, etc. Inflammation of the airways, continuous changes in their structure, epithelial cell damage, hypertrophy, and angiogenesis are other features [100]. Unfortunately, due to biological barriers, low drug bioavailability, and related safety issues, the effect of drug treatment alone in treating asthma is not yet satisfactory.

Magnetic nanoparticles are of great interest for site-specific drug delivery to the lungs. Dames et al. reported that superparamagnetic iron oxide nanoparticles (SPIONs), in combination with a target-directed magnetic gradient field, could target aerosol delivery to specific lung regions [101]. Soluble interleukin-17 receptor C (IL-17RC) protein is a common receptor subunit of IL-17 and IL-17F. It can act as a decoy receptor for IL-17 and IL-17F, inhibit downstream signal transduction, and ultimately prevent various inflammatory diseases [102]. Lv et al. used chitosan nanoparticles loaded with recombinant protein IL-17RC (rIL-17RC) to investigate its effect on allergic asthma model mice via intranasal administration. The results showed that intranasal administration of chitosan nanoparticles could significantly inhibit mucus secretion and airway inflammatory cell infiltration and reduce IL-4, IL-17, and IL-17F levels in bronchoalveolar fluids. Therefore, respiratory delivery of receptor proteins such as IL-17RC may be an attractive asthma intervention [103]. Curcumin is a well-known anti-inflammatory agent, but low bioavailability limits its efficacy. Gopalan et al. used nanoemulsion and microsuspension to explore pulmonary drug delivery of aerosolized curcumin. The in vitro nebulization performance of the nanoemulsion was better than the suspension formulation and was independent of drug concentration, while the performance of the suspension was dependent on the drug concentration. Furthermore, the nanoemulsion formulation was nontoxic at the curcumin doses used in the genotoxicity studies. Formulation performance and genotoxicity results suggest these formulations are suitable for further inhalation studies in animals and humans [104].

#### 4.4.3. Treatment of Acute Lung Injury/Acute Respiratory Distress Syndrome

Acute lung injury (ALI) or acute respiratory distress syndrome can lead to life-threatening respiratory failure. Despite significant advances in intensive care treatment and organ support techniques, mortality from ALI remains high at 30–40% [105]. Nanotechnology may provide new insights into ALI pharmacotherapy. Based on the abilities of lung targeting and ALI pathophysiology, various nanomedicines with different structures and functions have been developed [106].

In an inhalation treatment, Merckx et al. formed hybrid nanoparticles using nano-dextran nanogels loaded with siRNA as the core and the clinically used pulmonary surfactant Curosurf^®^ as the shell [107]. They found that surfactant protein B (SP-B) is a potent siRNA delivery enhancer. Prior to nanogel coating, SP-B was inserted into a simplified phospholipid mixture for improved siRNA delivery. The effect was observed in vitro (a lung epithelial cell line) and in vivo (a murine acute lung injury model). More importantly, the prepared nanoformulation exhibited low in vivo toxicity and could be efficiently taken up by resident alveolar macrophages, the primary target cell type for treating inflammatory lung diseases. This study demonstrated that endogenous protein SP-B could intracellularly enhance siRNA delivery and provides directions for designing nanoformulations for siRNA inhalation therapy. In terms of biodegradability and biocompatibility, poly (lactic-co-glycolic acid) (PLGA) is the most used polymer [108]. Ruthenium red-loaded PLGA nanoparticles were administered by inhalation in a ventilator-induced lung injury moDeliv. It was found that this nano drug reacted with alveolar macrophages as well as capillary endothelial cells, blocked calcium signaling, and inhibited vascular permeability in an in vitro ventilator perfusion study [109].

#### 4.4.4. Treatment of Lung Cancer

Over the past few decades, lung cancer has become the most common cancer in men and the second most common cancer in women worldwide, killing more than breast, prostate, colorectal, and brain cancers combined [110]. Projections suggest that worldwide deaths from lung cancer will continue to grow and are expected to exceed three million by 2035 [111]. Surgical resection, radiotherapy, and chemotherapy are currently the main treatments for lung cancer. However, non-selective drug distribution systemic toxicity, chemoresistance, and metastasis lead to treatment failure. In contrast, pulmonary drug delivery ensures localized drug delivery to the lung.

In vivo studies have found that nanoparticles accumulate at tumor sites after intravascular administration due to leaky tumor vascular structures [112]. These properties make nanoparticles a very attractive carrier for lung cancer treatment [113]. Azarmi et al. developed polysorbate 80-coated nanoparticles loaded with doxorubicin (DOX) to treat lung cancer and then incorporated the nanoparticles into inhalable carrier particles by spray freeze drying technology. A dry powder inhaler could deliver carrier particles containing DOX nanoparticles [114]. Compared with free DOX, DOX-loaded nanoparticles showed enhanced cytotoxicity in a concentration-dependent manner. The activity of DOX-loaded nanoparticles is enhanced as the nanoparticles are more easily internalized through the endocytic mechanism. Inhaled nanoparticles can be used to deliver anticancer drugs for local treatment to avoid systemic side effects [113]. Hitzman et al. reported the pulmonary delivery of 5-fluorouracil (5-FU) in a lipid-coated nanoparticle system to a hamster moDeliv. The pharmacokinetics of nanoparticles and a total 5-FU in lung, trachea, larynx, esophagus, and serum were studied [115]. Efficient local targeting and sustained effective concentrations of 5-FU were found to be achieved at the intended tumor site. The results suggest that 5-FU-containing lipid-coated nanoparticles can be used to treat lung squamous cell carcinoma.

Non-small cell lung cancer (NSCLC) is a global disease for which treatment options are still limited, and drug resistance of cancer cells and non-targeting of therapeutics are current significant obstacles. Parvathaneni et al. encapsulated pirfenidone (PFD) in a cationic liposomal carrier for NSCLC therapy. The potential of inhalable liposome-loaded pirfenidone in the treatment of NSCLC was assessed by colony formation, cell migration, apoptosis, and angiogenesis assays, but further clinical studies were needed to determine its efficacy [116]. Bedaquiline (BQ) is an FDA-approved anti-tuberculosis drug that has shown promising anti-cancer efficacy. However, poor aqueous solubility limits its delivery through the lungs. Patil et al. developed inhalable BQ-loaded cubosome (BQLC) nanocarriers using a solvent evaporation technique to treat NSCLC. The positively charged BQLCs were able to rapidly internalize cells and significantly inhibited the metastatic activity and colony formation of A549 cells compared to free BQ. This was the first study to explore the potential of cubosomes as inhalation therapy of BQ, providing preliminary evidence for the clinical application of the developed drug delivery system [117].

#### 4.4.5. Treatment of Idiopathic Pulmonary Fibrosis

Idiopathic pulmonary fibrosis (IPF) is a chronic progressive interstitial lung disease that affects more than three million people worldwide, with an average life expectancy of 3–5 years after diagnosis if untreated [118]. The pathogenesis of IPF involves recurrent microinjury of the alveolar epithelium in genetic predisposition, followed by an aberrant reparative response characterized by excessive collagen deposition. Despite being approved for the treatment of IPF, pirfenidone and nintedanib are associated with limited effectiveness in preventing disease progression and improving quality of life and have also been associated with tolerability issues [119].

Current IPF treatments are mainly administered orally. Inhalation therapy is already the mainstay of treatment for obstructive pulmonary diseases such as asthma and COPD but has been poorly executed in IPF [120]. A recent study using radio-labeled salbutamol confirmed that inhalation administration is feasible in IPF but is affected by fibrosis severity and particle size [121]. A study of patients with advanced IPF suggests that by combining N-acetylcysteine inhalation and oral pirfenidone, the rate of decline in forced vital capacity might be reduced and progression-free survival improved compared to pirfenidone alone [122].

### 4.5. Therapy for Systemic Diseases

The nasal mucosa has rapid blood flow, a highly vascularized epithelial layer, and a significant absorption area. These characteristics offer many advantages for introducing drugs into systemic circulation. The nasal route has been widely studied for the systemic administration of therapeutic agents and has been clinically used, for example with hormones and vaccines. Moreover, research on the lungs as an entrance for systemic drug delivery has been conducted for decades. Due to its large surface area of about 70–100 m^2^, permeable epithelium, and high perfusion properties, the respiratory mucosa is one of the best targets for biopharmaceutical uptake [123].

#### 4.5.1. Treatment of Diabetes

Diabetes is a complex chronic endocrine disease characterized by elevated blood glucose levels and a risk of life-threatening health problems [124]. Insulin is used to maintain blood glucose levels, but peptides are unstable in the gastrointestinal environment. It is often administered through subcutaneous injections. The subcutaneous route has shown low patient compliance due to frequent administration and injection site pain [125]. Other routes for administration, such as intranasal and inhalation, are considered alternatives to the subcutaneous route [126].

Desmospray is a solution for a nasal application containing desmopressin. Oral desmopressin showed minimum absorption from the gastrointestinal tract, and its bioavailability was only 0.08–0.16%, while the absorption from the nasal mucosa improved to 10–20% [127]. Therefore, intranasal administration of desmopressin may be used for certain diabetic complications. CP024 is a nasal powder formulation containing the human growth hormone. In Phase 1 clinical trials, CP024 was shown to induce insulin-like growth factor-1 to the same level as a subcutaneous injection of marketed products [128]. Several intranasal insulin formulations highlight the advantages of utilizing nasal drug delivery. For instance, Nas-tech Pharmaceuticals had successfully developed an intranasal insulin formulation with rapid action and bioavailability of 17–28%. The formulation was comparable to intravenous insulin, with safety and a shorter time to reach maximum concentrations in the body [129]. In another trial, glucagon-like peptide-1 was prepared as a powdered intranasal formulation administered as a nasal spray and tested blood glucose levels at specific times. Compared with subcutaneous administration, the bioavailability of the drug was 2.7%, and insulin levels increased with increasing doses. The preparation did not show any apparent toxicity or adverse drug reactions, few patients experienced side effects such as nausea and discomfort, and the drug and route of administration were proven safe [130].

For pulmonary drug delivery, in 1925, Gänsslen first reported the blood-glucose-lowering effect of inhaled insulin in five subjects with diabetes [131]. In 1989, scientists discovered that after the instillation of human growth hormone into the trachea, it was detected in the systemic circulation of rats [132]. These findings indicate that the pulmonary epithelial barrier is relatively permeable to macromolecules. Therefore, pulmonary drug delivery is considered an essential route for systemic administration. Inhaled human insulin is ultra-rapid-acting insulin approved by the FDA [133]. It can rapidly reach systemic circulation and attains the maximum plasma concentration within 15 min, much earlier than injectable insulin (i.e., 1 h).

These studies represent the exploration of using the respiratory route of administration to treat diabetes.

#### 4.5.2. Treatment of COVID-19

It is generally known that the nasal route for mucosal vaccination may induce a systemic and mucosal immune response [134]. Given the potential and current knowledge of mucosal delivery routes, many promising mucosal vaccine candidates have entered the clinic in the past few years. Designed to establish active immunity against influenza-related diseases in people aged 2 to 49 years, FluMist^®^ Quadrivalent blends four monovalent bulk influenza viruses to be delivered by nasal spray [135]. In infections where SARS-CoV-2 and other viruses invade mainly through the nasal mucosa, nasal vaccines are superior. In accordance with the WHO COVID-19-Landscape of novel coronavirus candidate vaccine development worldwide, there are 340 vaccine candidates in development, 145 of which are in clinical trials [136]. Of the 145 SARS-CoV-2 vaccines in the clinical phase, eight are designed for intranasal administration, two are designed for intranasal or intramuscular administration, and one is designed for both intranasal and intramuscular administration (Table 2).

Most vaccine candidates currently being tested in clinical trials are administered by injection [137]. Such liquid vaccines require a tightly regulated cold chain infrastructure, making it challenging to ensure large scale vaccination campaigns and requiring high costs. Producing lyophilized vaccines solves the stability problem of liquid formulations, but it still must be reconstituted before administration, so it does not eliminate the disadvantages of injections. Inhalable vaccines may solve this problem. In a clinical trial planned by the Imperial College London and the University of Oxford, the effectiveness of a viral vector-based vaccine will be assessed [138]. The vaccines will be given as aerosols to a small group of healthy volunteers in three escalating doses. There is potential for this study to provide valuable insight into the effectiveness of inhaled SARS-CoV-2 vaccines. In an inventive study by Lam and colleagues [139], mRNA was successfully dried using spray drying and spray freeze drying. These two techniques have been reported to have relatively high yields and can also be successfully used to create powders suitable for in vivo pulmonary administration. A viral spike glycoprotein binds to the angiotensin-converting enzyme 2 (ACE 2) receptor on the surface of host cells to facilitate the entry of SARS-CoV-2 into the host. Nanobodies, such as Nb11-59 [140] and Ty 1 [141], are designed to target the receptor binding domain (RBD) and prevent virus entry. These nanobodies exhibit effective virus neutralization activity in vitro and remain stable during nebulization. Inhaled nanobodies are expected to be an antiviral therapy against COVID-19 virus infection.

### 4.6. Challenges and Perspective

There are many advantages to using the nasal route to deliver drugs, but there are also some limitations. Several factors limit drug absorption and bioavailability in the nasal mucosa, including low membrane permeability for hydrophilic molecules with high molecular weights, a small volume of application, mucociliary clearance, mucus barrier, and the enzymatic environment [142]. Vaccinations applied to the mucosa may produce dilution, inactivation, or blocking of antigens by nasal secretions, enzymes, or epithelial barriers [143]. The retention time of formulations on the nasal mucosa may be limited by normal defense mechanisms, such as mucociliary clearance and ciliary beating. As a result, nasal epithelial cells are unable to efficiently uptake soluble antigens [127]. In addition, the nasal route is not ideal for delivering high molecular weight compounds, so large doses of the vaccines are required to achieve optimal immunity [144]. Nasal vaccines must therefore be specially formulated and optimized to ensure a positive immune response and prevent side effects such as local irritation. Moreover, it may be necessary to use adjuvants to increase the immunogenicity of nasal vaccines and deliver them to mucosal tissues.

Additionally, despite the advantages of pulmonary drug delivery, some critical factors need to be addressed. While the mucosal immune system protects the airways from pathogen invasion, excessive immune responses may worsen the condition of the lungs and cause enhanced antibody-dependent respiratory disease [137]. Studies have confirmed the idea that the immune system is overreacted in COVID-19 patients, showing that severe cases are associated with higher IgA titers [145]. Therefore, in evaluating inhaled vaccines, the potential role of IgA in adverse reactions, such as antibody-dependent cytotoxicity and antibody-dependent enhancement, should be considered. A second challenge to implementing pulmonary drug delivery is that animal models are often used to perform in vivo studies that require active drug administration, because of their inability to perform the required inhalation maneuvers [146]. It is also challenging to achieve adequate dispersion and deposition profiles with the most used in vivo delivery devices for pulmonary drug delivery. As a result, in vivo studies may not accurately reflect a patient’s condition. Considering their respiratory tract anatomy and their immunological response toward pathogens, vaccine candidates should be tested on animals with respiratory tract anatomy and immune responses to infections that are most like those found in humans [147].

While there are still some problems with respiratory administration, for the delivery of certain drugs and vaccines for specific purposes, the nasal and pulmonary routes may be as good or better than injections. There are two main challenges to respiratory delivery. The first is holding the system on the mucosal site for a sufficient time. Extended retention time can be achieved by mucoadhesive polymers (e.g., gelatin, mucin, chitosan, etc.) to ensure drug bioavailability in systemic circulation. Different mucosal sites offer specific similarities and differences from one another, and thus applicability varies according to the requirements of a particular treatment. Another challenge is improving the delivery system’s permeability, either by using penetration enhancers or by preparing advanced nanocarrier systems. Today, advances in nanotechnology, imaging, and drug delivery devices are driving rapid developments in drug delivery [134]. Recent advances in nanotechnology have driven the development of a broad spectrum of nano system carriers with the potential for nasal and pulmonary drug delivery [148]. A wide variety of drug delivery systems have been developed utilizing natural, synthetic, and semi-synthetic polymers to enhance mucoadhesion and prolong the residence time of the formulation at the site of application [149]. These systems prevent biodegradation and facilitate the passage of therapeutic molecules across biological barriers for efficient delivery to the disease site. We expect a significant increase in the number of formulations approved for nasal and pulmonary drug delivery.

## 5. Conclusions

In conclusion, by reviewing publications over the past two decades, this scientometric statistical analysis provided a visualized display of respiratory delivery research, including publication trends and keyword analysis. It revealed research hotspots and directions for respiratory delivery. Nasal and pulmonary drug delivery are the leading research direction in respiratory delivery, which can be used for local treatment (e.g., brain and lung diseases) and systemic therapy. High-frequency keywords related to respiratory delivery include nanoparticles, BBB, chitosan, insulin, asthma, and COPD, which are relevant to the two drug delivery methods. The application of respiratory delivery was deeply analyzed and discussed from three aspects: CNS disorders, lung diseases, and systemic diseases. Advances in formulation technology and drug delivery devices are driving rapid developments in drug delivery. Notably, recent advances in nanotechnology have driven the development of a broad range of nano system carriers. Of course, some scientific issues for respiratory delivery still require further research, such as local irritation after intranasal administration and the applicability of animal models. We expect a significant increase in approved formulations for respiratory delivery shortly. These findings reveal the current research hotspots and provide the foundation for further research on respiratory delivery.

## Figures and Tables

**Table 1:** Some of the FDA-approved inhalation products [89].

Brand Name	Drug	Indication	Manufacturer	Approval Year
Qvar Redihaler^®^	Beclomethasone dipropionate	Asthma	Norton Waterford	2017
Bevespi Aerosphere^®^	Glycopyrronium, Formoterol fumarate	COPD	Astrazeneca Pharms	2016
Asmanex^®^ HFA	Mometasone furoate	Asthma	Merck Sharp Dohme	2014
Duaklir Pressair^®^	Aclidinium bromide, Formoterol fumarate	COPD	Circassia	2019
Trelegy Ellipta^®^	Fluticasone furoate, Umeclidinium, Vilanterol	Asthma, COPD	Glaxosmithkline	2017
Airduo Respiclick^®^	Fluticasone propionate, Salmeterol xinafoate	Asthma	Teva Pharm	2017
Utibron^®^	Glycopyrrolate, Indacaterol maleate	COPD	Sunovion Pharms Inc	2015
Stiolto Respimat^®^	Tiotropium bromide, Olodaterol	COPD	Boehringer Ingelheim	2015
Spiriva Respimat^®^	Tiotropium	COPD	Boehringer Ingelheim	2014
Striverdi Respimat^®^	Olodaterol hydrochloride	COPD	Boehringer Ingelheim	2014

**Table 2:** COVID-19 nasal vaccine candidates in clinical development [136].

Vaccine Code/Name	Vaccine Type	Developer	Doses	Delivery Route	Development Stage	
DelNS1-2019-nCoV-RBD-OPT1	Viral vector (Replicating)	University of Hong Kong Xiamen University Beijing Wantai Biological	2 Day 0 + 28	IN	Phase 3	ChiCTR2100051391
COVI-VAC	Live attenuated virus	Codaginex Serum Institute of India	1–2 Day 0 or Day 0 + 28	IN	Phase 3	ISRCTN15779782
CIGB-669	Protein subunit	Centre for genetic Engineering and Biotechnology (CIGB)	3 Day 0 + 14 + 28 or Day 0 + 28 + 56	IN	Phase 1/2	RPCEC00000345
ChAdOx1-S-(AZD1222)	Viral vector (Non-replicating)	University of Oxford	1–2 Day 0 + 28	IN	Phase 1	NCT04816019
Razi Cov Pars	Protein subunit	Razi Vaccine and Serum Research Institute	3 Day 0 + 21 + 51	IM and IN	Phase 3	IRCT20210206050259N3
BBV154	Viral vector (Non-replicating)	Bharat Biotech International Limited	1 Day 0	IN	Phase 1	NCT04751682
MV-014-212	Live attenuated virus	Meissa Vaccines, Inc.	1 Day 0	IN	Phase 1	NCT04798001
Live rNDV vector vaccine	Inactivated Virus	Laboratorio Avi-Mex	2 Day 0 + 21	IM or IN	Phase 2/3	NCT05205746
PIV5	Viral vector (Non-replicating)	CyanVac LLC	1 Day 0	IN	Phase 1	NCT04954287
NDV-HXP-S	Viral vector (Replicating)	Sean Liu, Icahn School of Medicine at Mount Sinai	1 Day 0	IN or IM	Phase 1	NCT05181709

Abbreviations: IN, intranasal; IM, intramuscular.

## Data Availability

Not applicable.

## Supplementary Materials

The following supporting information can be downloaded at: https://www.mdpi.com/article/10.3390/pharmaceutics14091974/s1.
