# Supplementary material for: A Review of Non-Invasive Drug Delivery through Respiratory Routes
Source: Pharmaceutics. 2022 Sep 19;14(9):1974. doi: 10.3390/pharmaceutics14091974 (PMC9506287; doi:10.3390/pharmaceutics14091974)
Supplement: Supplementary file 1 [file pharmaceutics-14-01974-s001.zip › pharmaceutics-1880451-supplementary.pdf]

Supporting Information

A Review of Non-Invasive Drug Delivery through Respiratory Routes

Yong-Bo Zhang [1,2,†], Dong Xu [1,2,†,*], Lu Bai [1,2], Yan-Ming Zhou [1,2], Han Zhang [1,2] and Yuan-Lu Cui [1,2,*]

[1] State Key Laboratory of Component-based Chinese Medicine, Research Center of Traditional Chinese Medicine, Tianjin University of Traditional Chinese Medicine, Tianjin 301617, PR China

[2] Haihe Laboratory of Modern Chinese Medicine, Tianjin 301617, PR China

* Correspondence: superdong1994@gmail.com (Dong Xu); ylcui@tjutcm.edu.cn (Yuan-Lu Cui).

† These authors contributed equally to this work.

S. Results

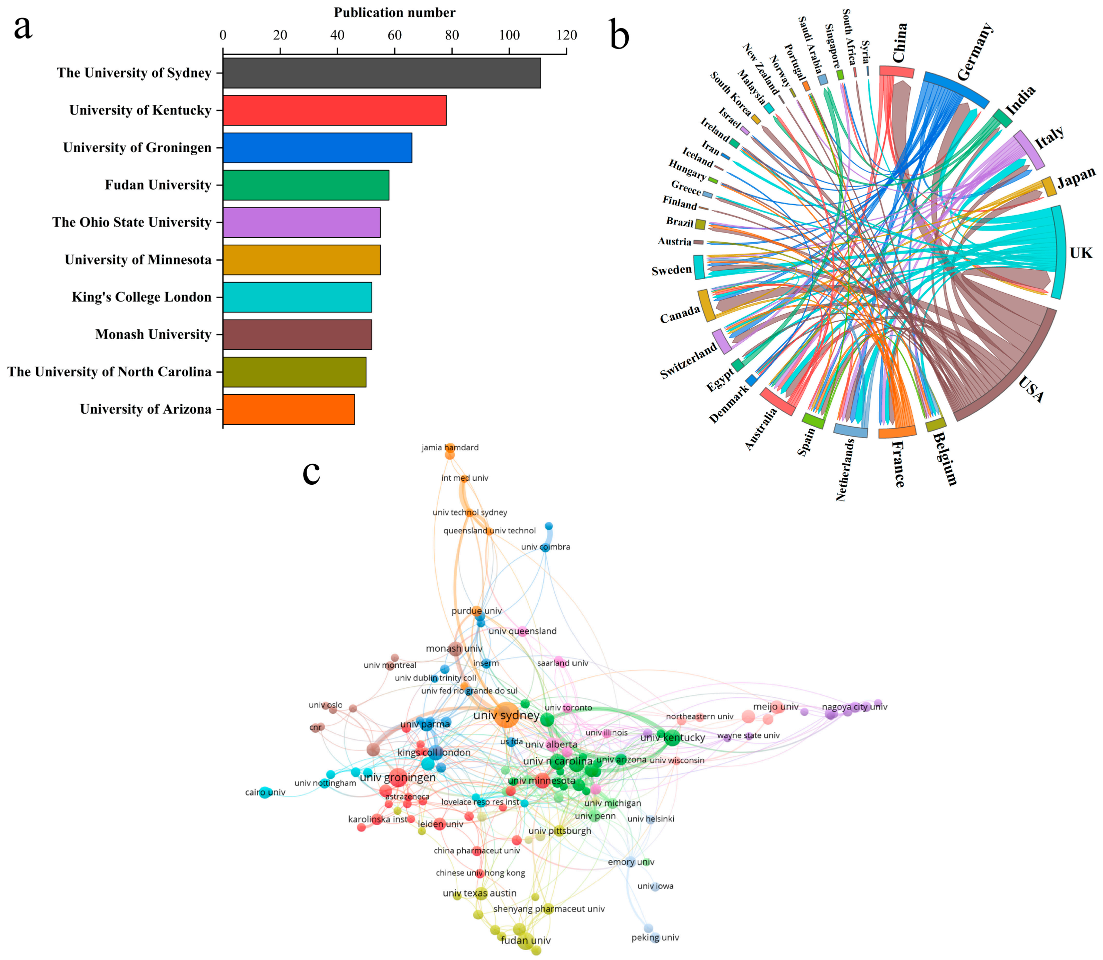

Figure S1. Scientometric study of respiratory delivery. **a**) The 10 most productive institutions. **b**) The cooperation relationships of countries/regions. **c**) The cooperation relationships of institutions. Node size represents the amounts of published articles. Lines of different colors show the cooperation between the two institutions.

Institutional Contribution and Country/Region Cooperation Analysis

There is some correlation between the number of articles published by an institution and its impact on this field of research. The University of Sydney is the most prolific institution, contributing 111 papers (Figure S1a). The USA has five institutions in the top 10, with the University of Kentucky (n = 78) ranking second. The ranking of USA institutions reflects its stronger scientific prowess in this field. University of Groningen (n = 66) in the Netherlands is ranked third in terms of publication number. The top 10 institutions also include Fudan University (n = 58) in China, King's College London (n = 52) in the UK, and Monash University (n = 52) in Australia.

Cooperative publications between countries reflect communication and research levels between the two countries. It is well known that cooperation among different countries and

institutions is an important driving force in promoting the development of science and technology. To this point, Figures S1b and S1c depict the partnership among countries and institutions, respectively, which demonstrate close cooperation among the various countries or regions. It is found that the USA accounts for a significant proportion of Chinese cooperative publishing. At the same time, there are many links between the USA and other countries, indicating that its cooperation with other countries is widespread (Figure S1b).

Journals and Highly-Cited Papers

The top 10 journals with the most publications are listed in Table S1. The top 10 journals published 762 papers on respiratory delivery, comprising 19.43% of the total. *International Journal of Pharmaceutics* published the most articles with 215 papers, followed by the *Journal of Pharmaceutical Sciences* with 72 papers and the *Vaccine* with 71 papers.

Table S2. lists the top 10 most cited articles, which are generally considered vital to the study of respiratory delivery. In particular, the most cited paper (756 citations) is a review titled Inhaling medicines: delivering drugs to the body through the lungs, published by *Nature Reviews Drug Discovery* in 2007. The next highly cited article (692 citations) is a review of intranasally administering to the central nervous system.

Table S1. Top 10 journals with the most publications.

Rank	Journal	Number of Publication	% of Total Publication	Impact Factor (2021)
1	International Journal of Pharmaceutics	215	5.48	6.510
2	Journal of Pharmaceutical Sciences	72	1.84	3.784
3	Vaccine	71	1.81	4.169
4	European Journal of Pharmaceutics and Biopharmaceutics	70	1.79	5.589
5	Journal of Controlled Release	70	1.79	11.467
6	Pharmaceutical Research	67	1.71	4.580
7	European Journal of Pharmaceutical Sciences	58	1.48	5.112
8	PLoS One	49	1.25	3.752
9	Expert Opinion on Drug Delivery	47	1.20	8.129
10	Drug Delivery	43	1.10	6.819

Table S2. The top 10 highly cited documents ranked by total citations.

Rank	Article title	Year	Total Citations	Journal Name	Journal Impact Factor (2021)
1	Inhaling medicines: delivering drugs to the body through the lungs	2007	756	Nature Reviews Drug Discovery	112.288
2	Intranasal Delivery to the Central Nervous System: Mechanisms and Experimental Considerations	2010	692	Journal of Pharmaceutical Sciences	3.784
3	Nasal drug delivery—possibilities, problems and solutions	2003	675	Journal of Controlled Release	11.467
4	Pulmonary drug delivery. Part I: Physiological factors affecting therapeutic effectiveness of aerosolized medications	2003	631	British Journal of Clinical Pharmacology	3.716
5	Delivery of insulin-like growth factor-I to the rat brain and spinal cord along olfactory and trigeminal pathways following intranasal administration	2004	613	Neuroscience	3.708
6	Intranasal delivery of biologics to the central nervous system	2012	547	Advanced Drug Delivery Reviews	17.873
7	Mechanisms of Actions of Inhaled Anesthetics	2003	536	New England Journal of Medicine	176.079
8	Formulation strategy and use of excipients in pulmonary drug delivery	2010	432	International Journal of Pharmaceutics	6.510
9	Once-Daily Single-Inhaler Triple versus Dual Therapy in Patients with COPD	2018	430	New England Journal of Medicine	176.079
10	Particle Engineering for Pulmonary Drug Delivery	2007	426	Pharmaceutical Research	4.580
